# In vivo continuous evolution of metabolic pathways for chemical production

**DOI:** 10.1186/s12934-019-1132-y

**Published:** 2019-05-14

**Authors:** Zheng Lin Tan, Xiang Zheng, Yinan Wu, Xingjin Jian, Xinhui Xing, Chong Zhang

**Affiliations:** 1 MOE Key Laboratory for Industrial Biocatalysis, Institute of Biochemical Engineering, Department of Chemical Engineering, Tsinghua University, Beijing, 100084 China; 2 School of Life Science and Technology, Tokyo Institute of Technology, Yokohama City, Kanagawa Prefecture, 226-8503 Japan; 3 Laboratory of Future Interdisciplinary Research and Science Technology, Tokyo Institute of Technology, Yokohama City, Kanagawa Prefecture, 226-8503 Japan; 4 Center for Synthetic and Systems Biology, Tsinghua University, Beijing, 100084 China

**Keywords:** In vivo continuous evolution, In vivo genotype diversification, Fitness-coupled selection pressure, Equipment maintaining continuous culture, Metabolic pathway evolution, Chemical bioproduction, Microbial cell factory evolution

## Abstract

Microorganisms have long been used as chemical plant to convert simple substrates into complex molecules. Various metabolic pathways have been optimised over the past few decades, but the progresses were limited due to our finite knowledge on metabolism. Evolution is a knowledge-free genetic randomisation approach, employed to improve the chemical production in microbial cell factories. However, evolution of large, complex pathway was a great challenge. The invention of continuous culturing systems and in vivo genetic diversification technologies have changed the way how laboratory evolution is conducted, render optimisation of large, complex pathway possible. In vivo genetic diversification, phenotypic selection, and continuous cultivation are the key elements in in vivo continuous evolution, where any human intervention in the process is prohibited. This approach is crucial in highly efficient evolution strategy of metabolic pathway evolution.

## Introduction

Nature is the best chemist that the precision of biosynthesis of chemical products are unmatched by conventional organic synthesis. However, wild type microbial cells are often evolved to maximise survival and growth in native habitat [1], resulting in low production, yield and titre to fulfil industrial requirements. These microbial cells can be retrofitted into highly effective chemical production factories through rational design approach. When the related knowledge is available and precise, rational design approach is an efficient tool of genetic modification. Furthermore, this approach provides a powerful tool to create a microbial cell factory from scratches. In the past decades, various natural and unnatural chemical products have been produced using rationally designed microbial cell factories (refer [2–4]). However, although metabolic engineers continue to reveal the relationship between carbon flux, genetic sequences and yield, our knowledge is still very limited, hindering us from precisely predicting the phenotypic outcome of genetic modification. This limitation has made knowledge-based rational design a cumbersome and time-consuming process. Extensive knowledge and intense works are required for limited improvement of production, yield or titre.

Nature has her way to optimise metabolic pathway efficiently. Evolution, as described by Darwin, is a continuous process of mutation and adaptation which, through diversification and natural selection, provides an opportunity for the survival of the fittest [5]. Darwinian evolution is also a solution to knowledge-free metabolic pathway modification provided by nature [6, 7]. However, in order to preserve essential genetic information over a long time, natural evolvability is extremely low [8, 9]. Furthermore, screening was impossible for unobservable phenotype, further reducing the discovery rate of microbial strains with desired properties [10]. Directed evolution is an in vitro process, developed to mimic the natural evolution at a higher rate, towards a defined goal. Successful demonstration of in vitro Darwinian evolution in 1967 [11], has inspired scientific community to envision evolution as an efficient methods to discover products with novel properties. Amplification of Qβ bacteriophage genomic ribonucleic acid (RNA) results in the shrinking of genome to 17% of its original size, with 15 times increases in replication rate, after 74 serial passages. Error prone (ep)-polymerase chain reaction (PCR) which has later been introduced, achieves in vitro random mutagenesis and selection by reducing the fidelity of PCR under various conditions [12]. Since then, directed evolution have been widely applied, not only in the optimisation of biological systems (review can be found in [13–17]), but also in forming novel biological functions [18, 19]. These examples have demonstrated great opportunities provided by evolution. However, human intervention is required in every step in in vitro directed evolution process. This limitation has become a bottleneck when attempting to optimise large, complex systems, as there are too many combinations in the combinatorial space, rendering in vitro directed evolution approaches unfit for deep mesoscale optimisation.

**Fig. 1:**
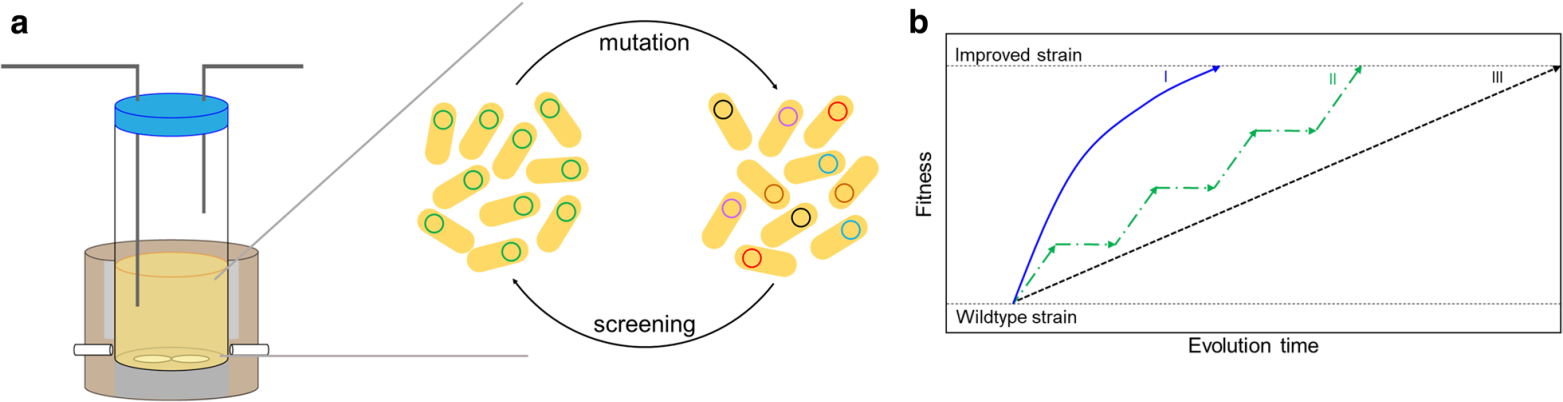
Illustration of in vivo continuous evolution. **a** General concept of in vivo continuous evolution as an uninterrupted Darwinian evolution occurs for an extended period of time in continuous culturing system. 3 major aspects, in vivo genotype diversification, fitness-coupled selection pressure and environment maintaining continuous culture are well integrated into a system. The mutation occurs endogenously and along with proliferation. **b** The conceptual difference between in vitro continuous evolution, in vivo evolution and in vivo continuous evolution. I represents in vivo continuous evolution which genotype diversification, natural mutation and selection are integrated. This process occurs as a smooth process without distinguishable plateaus as in in vitro continuous evolution; II represents in vitro continuous evolution dividing into genotype diversification and selection; while III represents in vivo evolution showing a process in which mutation accumulates to form a desired strain

Back in nature, Darwinian evolution cycle is a natural process without any form of human intervention. In our perception, an ideal Darwinian evolution enabling efficient evolution is supported by 3 major aspects (1) in vivo genotype diversification, (2) fitness-coupled selection pressure and (3) environment maintaining continuous culture, well-integrated in a system. Based on the above-mentioned perception, in vivo continuous evolution can be considered as a system with two main characteristics, (1) endogenous mutagenesis and (2) occurrence of mutagenesis along with proliferation (Fig. 1). This nature-tailored automated process is the key to realise efficient evolution of organisms. Microbial cells factories are continuously mutagenised and selected in a continuous culturing system to induce rapid evolution (Fig. 1a). Although longer evolution time is required for in vivo evolution compared to in vitro evolution to obtain an improved targeted strain, human intervention is not required for in vivo evolution when an automated continuous culturing approach is employed. This automated continuous cultivation approach has freed the labours from benchwork, hence, increasing the time efficiency of each step in experiment. However, the involvement of intense labour in in vitro evolution, and longer time-consumption in in vivo evolution due to its random mutagenic nature have rendered them impractical for deep mesoscale optimisation of large, complex pathway in a short time. Further improvement to accelerate the process is made by coupling genotype diversification, natural mutation and selection into a single process (Fig. 1b), known as in vivo continuous evolution. With its advantage over directed evolution, in vivo continuous evolution is becoming an important tool to evolve large, complex metabolic pathways for chemical production [20]. In this review, we will highlight the latest developments of each aspect in in vivo continuous evolution including in vivo genotype diversification, fitness-coupled selection pressure and equipment maintaining continuous culture. We will present a systematic review of recent advances in in vivo genotype diversification technology and the comparisons of these technology covering modified natural mutagenesis system, plasmid-targeted mutagenesis system, genome-targeted mutagenesis system and recombination-based mutagenesis system. Next, we will analyse fitness-coupled selection pressure, covering natural and artificial metabolite production/cell fitness coupling for phenotypic selection. As a system mimicking natural continuous evolution mechanism, we will also review equipment maintaining continuous culture, including flask, chemostat, turbidostat, microfluidic and droplet-based continuous culturing system. Then, we will introduce some cases successfully applying in vivo continuous evolution in improving metabolic pathway of microbial cell factories. Finally, we will evaluate importance of each technology in the integrated system of in vivo continuous evolution, and their inter-relationship to provide a comprehensive and quantitative understanding of these technologies in in vivo continuous evolution of metabolic pathways for chemical production.

## In vivo genotype diversification

**Fig. 2:**
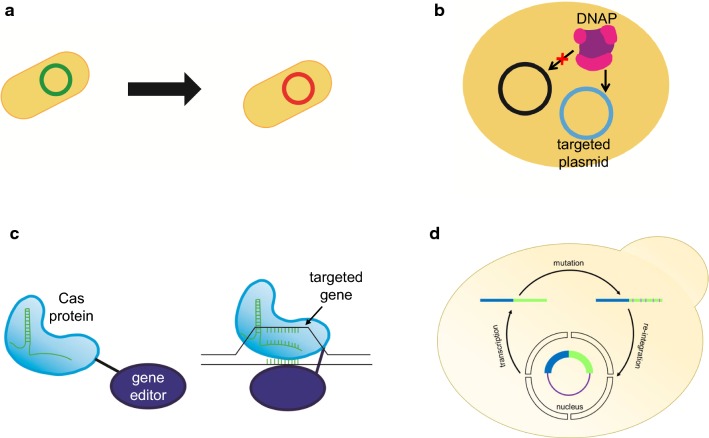
In vivo genotype diversification. **a** Modified natural mutagenesis system. Random mutagenesis with higher mutation rate is induced with mutators. **b** Plasmid-targeted mutagenesis system. DNA plasmid-DNAP pair is designed such that the ep-DNAP mutates only the targeted plasmid. **c** Genome-targeted mutagenesis system. A gene editor is bind to a targeting protein, usually a Cas protein to mutate only a specific locus in genome. **d** Recombinase targeted mutagenesis system. Native system in microbial cells is used to recombine or re-integrate mutated gene into the plasmid

Genetic diversification is the first step in in vivo continuous evolution to generate a diverse mutant library before selection to maximise the possibility to obtain an optimal strain. Natural mutation is an example of in vivo random mutagenesis [8, 9]. However, the natural mutation rate is very low, which is around 2 to 5 in 10 billion base pairs per generations for most species [8, 9]. Modern molecular biology has enhanced the understanding of mutagenetic mechanism. Table 1 summarised in vivo genotype diversification methods developed based on modern molecular biology, while Fig. 2 illustrate the mechanism of each category, which will be introduced as the followings. These methods continuously trigger in vivo mutagenesis throughout continuous cultivation process, while selection occurs continuously. Simultaneous occurrence of in vivo mutagenesis and selection in an equipment maintaining continuous culture results in a smooth evolution curve, accelerating the process to obtain strains with desired properties.

**Table 1:** In vivo genotype diversification strategies for in vivo continuous evolution

Type	Methods	Targetability	Window size	Mutation rate, nucleotides^−1^	Trackability	Description	Ref
Modified natural mutagenesis system	*Pol*III	No	N/A	≈ 10^−10^	No	Introducing error-prone dnaQ into the system to increase mutation rate	[23]
	*Pol*I	Yes	650 bp	≈ 8.14 × 10^−4^	No	Targeted gene is transferred to the preferential region of low fidelity variant of *Pol*I to achieve error-prone transcription. The mutation rate can be enhanced by the absence of *mutL* and *mutS* system. However, this system is distance dependence	[27]
Plasmid-targeted mutagenesis system	OrthoRep	Yes	3.7 kbp	≈ 10^−5^	No	Orthogonal DNA plasmid-DNA polymerase pair extranuclear replication system is used. Another TP-plasmid containing targeted gene is prepared to be targeted by ep-DNAP to increase the mutation rate on the targeted region while maintaining the nature mutation rate of the plasmid containing all the essential genes	[36–38]
Genome-targeted mutagenesis system	TaGTEAM	Yes	20 kbp	≈ 10^−7^	No	Targeting the binding site of DNA glycosylase (MAG1) and DNA binding protein (tetR) with a mutation generation system through ep-HR by resectioning and ep-*Pol* ζ	[39]
	EvolvR	Yes	350 bp	≈ 10^−5^–10^−6^	Yes	Point targeting of targeted gene using CRISPR-nCas9, and mutate the targeted gene with DNAP *Pol*3 M attached to CRISPR-nCas9	[41]
	Base editing	Yes	18–23 bp (100 bp for CRISPR-X)	N/A	Yes	Point targeting of targeted gene using Cas9 protein, while editing base using base editor attached to Cas9 protein	[42–59]
Recombination-based Mutagenesis System	SCRaMbLE	Yes	N/A	loxPsym dependence	Yes	Insertion of loxPsym after the stop codon of non-essential gene, and trigger chromosomal rearrangement using Cre recombinase	[63, 65–69]
	Retron	Yes	5 kbp	≈ 6.67 × 10^−3^	Yes	The targeted gene labelled with retrotransposon recognition label is transcribed, followed by reversed transcription to generate specific mutation on gene	[70, 72, 73]

Targetability indicates the ability to target a specific locus in gene; window size indicates the size of targetable window; trackability indicates the trackability of mutation induced

### Modified natural mutagenesis system

Modified natural mutagenesis is a system based on modification of nature-existing mutation mechanism, particularly, mutator isolated from highly mutating bacteria (Fig. 2a) [21, 22]. These mutators, when analysed carefully, proven to unidirectionally transverse certain nucleotides, i.e., *mutT* unidirectionally transverse A∙T bases to C∙G bases [21] (A detail review can be found in [23]). As most of the components used in this system are natural, the operations are relatively simple, with a slightly higher mutation rate compared to natural mutation. For instance, polymerase (pol) III ε-subunit dnaQ [24], increase the mutation rate of host genome by 150 times. Besides, the mutation rate for a commercially available *Escherichia coli* competent cell XL1-red with deactivations on its proofreading and repair enzymes, *mutD*, *mutS*, *mutT* is limited to 10^−6^ base^−1^ [25]. However, when applying modified natural mutagenesis system, it is important to note that intolerance might occur due to host mutation. Moreover, accumulation of host genome mutation might result in cytotoxicity and reduction in genetic stability [26].

### Plasmid-targeted mutagenesis system

Plasmid-targeted mutagenesis system was introduced to confine mutagenesis within the targeted plasmid, thus preventing mutation in host genome. The first in vivo targeted plasmid mutagenesis system is demonstrated with the utilisation of ep deoxyribonucleic acid (DNA) polymerase I (*Pol*I). Targeted gene is placed in *Pol*I preferential region to be replicated by a low fidelity variant of *Pol*I [27] (refer [28–31] for the functions of *Pol*I). However, the replication rate was low. In the demonstration on LacI mutagenesis, 57 mutants were generated per million cells after 30 generations. Although the mutation rate is 5000-fold higher compared to background mutation, the frequency is still considered low overall. Mutation rate was further enhanced by conjunction with the absence of *mutHLS* system. Another 20-fold increase in the absence of *mutS* system, and 40-fold in the absence of *mutL* system was achieved [27]. However, the mutation rate in this system is distance dependence. The mutation rate drops by approximately 6 to 20 times when it is located far from the colE1 origin of replication, which is the targeted site of *Pol*I [32]. On the other hand, another approach to improve *Pol*I-induced mutagenesis by introducing point mutations in three structural domains, Ile^709^ and D424A in motif A [33], and A759R in motif B (O helix) [34] which govern the fidelity in DNA *Pol*I is proposed. The mutation rate is increased by 80,000-fold [35]. However, due to the uncontrollability of polymerase in cell, there is a risk of ep-*Pol*I scattering and reduction of polymerase-plasmid orthogonality, which will result in the targeting on undesired fragments, causing a mutation on host genome, as high as it is achieved in the targeted region.

Another method called OrthoRep was proposed to avoid the limitation in *Pol*I by using heterologous plasmid-polymerase pair, exploiting *Kluyveromyces lactis* cytoplasmic plasmid system [36–38]. This system is an orthogonal DNA plasmid-DNA polymerase pair extranuclear replication system in yeast. In this replication system, there is a terminal protein (TP)-plasmid containing targeted gene, and another plasmid containing all the essential genes. Targeted mutagenesis with strict orthogonality of TP-DNA polymerase (DNAP) autonomous replication process is achieved by engineering an ep-DNAP to target the TP-plasmid, resulting in rapid mutation of the targeted plasmid (Fig. 2b). The contrast between targeted (3.5 × 10^−8^) and global (10^−10^) mutagenesis was achieved by the nature of p1 replication initiation mechanism and spatial separation from nuclear DNA [36].

Both *Pol*I and OrthoRep are limited to specific host cell due to their unique mechanism. Although cross-transferring of the technology was not possible, each has provided a tool to modify their respective system, covering the two main types of microbial cell species which are commonly used as microbial cell factories. However, the complicated setup of these systems has limited the application. A well-designed system is required before mutagenesis can be induced. Furthermore, mutation in mutagenesis-inducing plasmid can lead to the loss of orthogonality of plasmid-polymerase pair, resulting in the failure of targeting effect.

### Genome-targeted mutagenesis system

With the emergence of gene-targeting technologies, a more precise system targeting specific locus on gene rather than plasmid is developed as genome-targeted mutagenesis system. An example to this system is targeting glycosylases to embedded arrays for mutagenesis (TaGTEAM) [39]. TaGTEAM is designed to target the binding site of DNA binding protein, TetR. This technology depends on resectioning and ep-*Pol* ζ to generate mutation through ep homologous recombination (HR). Although 800-fold of elevation in point mutation within 20 kbp region was developed, particular attention is required on the fact that 24.5% deletion rate has also been observed. This can lead to the loss in important genetic information in the targeted fragment.

The invention of clustered regularly interspaced short palindromic repeats (CRISPR) genome editing technology [40] is a game changer to in vivo genetic diversification technology. CRISPR associated (Cas) protein was coupled with a mutator protein, offering synergy advantages of both systems. Cas protein offers precise targeting mechanism; while high mutation rate is realised with mutator proteins such as *Pol*I and base editing enzymes (Fig. 2c). These genome editing tools function on only a strand of double-stranded DNA. Hence, neither double strand break (DSB) will be induced, nor the system is HDR dependence, nor it requires a template. EvolvR [41] and base editing [42–44] are two examples to this category. EvolvR coupled CRISPR-nickase Cas protein 9 (nCas9) to ep-DNAP *Pol*I to combine the advantage of both systems, while preventing DSB by mutating RuvC nuclease domain in Cas9. For base editing, approximately 33 types of base editors, categorised into cytosine base editors and adenosine base editors are developed. These editors generally have 18 to 23 bp window size [42–58], with an exception of CRISPR-X having a 100 bp window size [59]. However, controversies remained with CRISPR-based system. Unlike other targeted mutagenesis platform, off-target mutagenesis is one of the greatest problems in CRISPR system [60–62].

### Recombination-based mutagenesis system

Along with genome-targeted mutagenesis system, another technology based on in vivo recombination has been developed at the same time for targeted mutagenesis. This technology was made possible by recent developments of in vivo recombinase expression system, or engineering microbial cell native retrotransposon element.

Synthetic chromosome rearrangement and modification by loxP-mediated evolution (SCRaMbLE) [63] utilising Cre/loxP recombination system was proposed for high-throughput mutagenesis (refer [64] for Cre recombinase). LoxPsym, a palindromic DNA sequence is inserted after stop codon of non-essential gene at a distance of 3 bp in the synthetic genome. With the presence of Cre recombinase, recombination among loxPsym sites occurs. LoxPsyms break up symmetrically, allowing random recombination of gene. An oestradiol switch was designed to control the production of Cre recombinase in the cell and Cre recombinase was designed to release only once in a lifetime to prevent multiple recombinations. To date, several variants of SCRaMbLE have been developed. Reporter genes were inserted into the circuit to distinguish those which have undergone SCRaMbLE [65]; design of red light activating SCRaMbLE [66]; activation by galactose in addition to oestradiol [67] and introduction of multiple pulses of Cre recombinase [68] to reduce recombinase activity. As an application of in vivo genotype diversification for chemical production, SCRaMbLEd yeast mutant with more than 2-fold increase in violacein and penicillin production were generated [69]. Diversification efficiency produced by SCRaMbLE is proportion to loxPsym inserted into the gene. Although diverse mutant library can be created by inserting more loxPsym into the chromosome, the diversification efficiency is also limited by the maximum concentration of loxPsym. Furthermore, SCRaMbLE induces deletions and inactivation of essential genes. More SCRaMbLE events might lead to less viability of SCRaMbLEd microbial cells.

Retron-based targeted mutagenesis is another approach to achieve high mutation rate with recombination approach. It is a mutagenesis approach utilising native retrotransposons in microbial cells which exhibit similar properties to single-stranded RNA (ssRNA) retroviruses. In *E. coli*, it depends on Ec86, the native retron in *E. coli* as core module to transcribe and reverse transcribe the content in ssDNA. The precision to target a homologous DNA region in chromosome depends on β recombinase (recβ) from bacteriophage λ [70], which is known for its single strain binding properties in λRed recombination [71]. On the other hand, in yeast [72, 73], retrotransposon-based element is the equivalent of retron. The targeted gene labelled with Ty1 retroviral recognition flank is transcribed, then reverse transcribed by Ty1 reverse transcriptase (Fig. 2d). This process generates specific mutation on gene. The mutated gene is then re-integrated into its locus by Ty1 integrase. Mutation rate as high as 1.5 × 10^−4^ base^−1^ at URA3 locus is achieved. This strategy provides a high mutation rate with high target specificity compared to other methods. Furthermore, the utilisation of yeast native retrotransposon has greatly reduced the risk to damage the host cell as in other methods. However, due to its dependence on retrotransposon Ty1, this method is limited to *S. cerevisiae* and *K. lactis*.

## Fitness-coupled selection pressure

Under normal conditions, microbial cells prefer not to use growth irrelevant chemical production pathway which may impose extra metabolic burden and reduce cell growth. However, we can design a microbial cell factory by directing microbial cells to use the production pathway of desired chemical via growth-production coupling. Enrichment of these desired cells can be achieved by selection. When microorganisms are transferred into environment with harmful selection pressure, e.g., β-lactam, they evolve β-lactamase metabolic pathway to destroy amide bond of β-lactam ring [74, 75]. Under natural selection, only the fittest microbial cells, i.e., those producing the most β-lactamase are able to survive the best. Furthermore, under normal culturing conditions where the nutrient supply is constant, microbial cells evolve to optimise their carbon source utilisation pathway to maximise their growth. The increase in scale fitness of *E. coli* by 1.8 times after 50,000 generations [76] in the *E. coli* long-term evolution experiment (LTEE) have hinted us on the potential in adaptive evolution under selection pressure to evolve microbial cell to optimise their stock utilisation pathway. These properties can be exploited for in vivo continuous evolution and have long been utilised in microbial cells evolution for chemical production. Table 2 shows the details of various fitness-coupled stress selection system, which will be introduced as the followings.

**Table 2:** Fitness-coupled selection pressure for in vivo continuous evolution

Type	Selection pressure	Principle	Products	Original yield or titre	Optimised yield or titre	Evolution time, generations	Ref
Natural metabolite production/cell fitness coupling	Hydrogen peroxide	Production of β-carotene as antioxidant to neutralise the oxidative stress of hydrogen peroxide	β-carotene	6 mg g^−1^ [dcw]	18 mg g^−1^ [dcw]	40	[79]
	LB medium	Coupling ATP and lactate production to growth by engineering lactate production route as the sole anaerobic NADH oxidation route	d-lactate	Maximum production: 865 ± 36 mmol l^−1^ Yield: 86%	Maximum production: 1071 ± 2 mmol l^−1^ Yield: 93%	34	[83]
	NBS medium with glucose and betaine (optional)		l-lactate	Maximum production: 1228 ± 31 mmol l^−1^ Yield: 95%	Maximum production: 1314 ± 48 mmol l^−1^ Yield: 98%	80	[85]
Metabolic evolution	NBS or AM1 medium with glucose	Coupling ATP and growth to alanine production and NADH oxidation	l-alanine	Maximum production: 181 mmol l^−1^ Yield: 81%	Maximum production: 1279 mmol l^−1^ Yield: 95%	123	[86]
	NBS medium with 9% xylose	D(–)-lactate hydrogenase fermentation pathway as the sole fermentative pathway coupled with the growth of strain	Ethanol	≈ 250 mM	≈ 950 mM	38	[87]
	NBS or AM1 medium with glucose	Coupling growth and glucose fermentation to NADH oxidisation pathway	Succinate	108 mM	699 mM	150	[88, 89]
			Malate	0 mM	313 mM		
Artificial metabolite production/cell fitness coupling	Nickel ion	Riboselector connecting phosphoenolpyruvate to oxaloacetate which is a precursor to lysine	l-lysine	0.0 g l^−1^	0.6 g l^−1^	12	[92]
	Sodium dodecyl-sulphate (SDS) for positive selection and colicin E1 for negative selection	Sensor-selector connecting a chemical sensor to a cognate promoting operator controlling selector and tolC toggle switch for reverse selection	Naringenin	1.69 mg l^−1^	61 mg l^−1^	60^a^	[90]
			Glucaric acid	0.05 mg l^−1^	1.2 mg l^−1^	5^a^	[90]
	Maltose	Maltose hydrolase is coupled to tryptophan sensor and the cell is cultured in a medium with maltose as a sole carbon source to couple cell growth to tryptophan production	l-trytophan	0.5 mg l^−1^ OD_600_^−1^	5 mg l^−1^ OD_600_^−1^	12	[95]
	N/A	Co-cultivation of auxotroph to couple the growth of secretor strain and sensor strain to amplify the difference in production level. 2-ketoisovalerate auxotroph is used as sensor strain, while lysine auxotroph is used as secretor strain	Isobutanol	1.8 ± 0.1 g l^−1^	9.4 ± 0.4 g l^−1^	N/A	[96]

^a^Number of cycles of multiplex automated genome engineering (MAGE) followed by toggled selection

### Natural metabolite production/cell fitness coupling

In most cases, organisms are able to evolve some properties to protect themselves from harms caused by selection pressure. Under selection pressure, microbial cells which produce more metabolite are able to proliferate and have a higher growth rate compared to low producing cells, hence outcompeting the low-producing cells in the culture after several dilution culture. This phenomenon can be observed in the regulation of gene expression in microbial cells under selection pressure using modern genetic analysis tools. When *E. coli* is grown in toxic level of ethanol, the expression of almost all genes in tricarboxylic acid (TCA) cycle, and genes related to glycine, glycine betaine, peptidoglycan, colanic acid and enterobactin synthesis are up-regulated to enhance ethanol tolerance [77]. It has also been found that *E. coli* lack of glutamate-cysteine ligase (*gshA*) gene, an important enzyme for the formation of γ-glutamyl cysteine production for glutathione (GSH) synthesis, evolved another GSH producing pathway from l-proline synthesis pathway, to protect the microbial cell from stressful conditions [78]. These results show that selection pressure is useful in in vivo continuous evolution of metabolic pathway when the cell fitness is coupled to desired products.

Wild type yeasts produce isopentenyl diphosphate (IDP) which is the natural precursor to carotenoid. This antioxidant is secreted to prevent the cells from being oxidised when exposed to oxidative stress. Evolution of β-carotene production pathway in *S. cerevisiae* with natural metabolite production/cell fitness coupling has successfully been demonstrated with an increase in β-carotene yield by 3-fold, to 18 mg g^−1^ [dcw] using periodic hydrogen peroxide shocking strategy [79].

Besides chemical stress, physical stress can also be used to increase the production of chemical products. Shinorine is a compound in mycosporine-like amino acids (MAAs) family, produced by marine microorganisms. This compound has an absorption maximum of 333 nm [80]. This property makes it an important ingredient in some sunscreen products. Biosynthesis of shinorine in microbial cell factory has been successfully demonstrated [81]. Although evolution has not been conducted in this study, the higher growth rate of shinorine producing cyanobacteria *Synechocystis* hinted that this pathway is evolvable when it is exposed to ultraviolet ray. Lack of nonribosomal peptide synthase (NRPS)/polyketide synthase (PKS) gene cluster in cyanobacteria [82] leads to the null effect in shinorine production in cyanobacteria exposed in ultraviolet ray. Using other type of microbial cell as host might result in the physical evolvability of shinorine production.

However, metabolite production/cell fitness coupling for chemical production does not always exist in nature. This method is limited to the pairs of various damaging source which can harm the microbial cell.

### Metabolic evolution

Metabolic evolution, a method using synthetic circuit to evolve microbial cells during fermentation process was proposed [83]. Essential cofactor recycling is coupled with the target pathway as the sole pathway to link the production of chemical products to the growth of microbial cells to induce evolution during fermentation. Sequential dilution was performed to enrich improved strain produced by evolution, and isolation was performed by streaking. In the earliest demonstration, lactate production pathway in *E. coli* was engineered as the sole anaerobic nicotinamide adenine dinucleotide hydride (NADH) oxidation route to couple ATP and lactate production to growth [83]. The production of various chemical products, such as d-lactate [83, 84], l-lactate [85], l-alanine [86], ethanol [87], succinate [88, 89] has been improved using metabolic evolution (details listed in Table 2). This technology involves not only metabolic evolution, but also synthetic pathway construction to couple cell fitness to metabolite production. Vast knowledge is required to enable evolution during fermentation process without the introduction of selection pressure. The difficulties in pathway design has limited the spreading of this technology. However, with the emergence of automated continuous cultivation technology with the ability to trace not only the production, but also cell growth in each vial, the benchwork involved in this system can be greatly reduced, transforming this system into a convenient evolution approach.

### Artificial metabolite production/cell fitness coupling

Although it is difficult to find a link between cell fitness and industrial-relevant chemicals in nature, the sensors for these compounds exist naturally. Synthetic biologists have engineered biosensor regulating antibiotic resistance gene into gene circuit, creating an artificial linkage of cell fitness to chemical production [90], mimicking the phenomenon of stress resistance adaptive evolution in wild type microbial cells. This is applicable to most chemical production pathway with the tools developed for synthetic biology.

Considering cell as a machine, the relationship between metabolite production and selection pressure can be understood as a sensor-actuator module in the machine. With an input of the concentration of chemical affecting cell fitness, the sensor transmits the signal to the actuator, producing an output of metabolite production. An RNA device called riboswitch is a module that fulfil this function (detail review can be found in [91]). A riboselector comprising a riboswitch and a selection module which act as a functional unit, is utilised in mutant screening. An increased in production of l-lysine, using nickel ion as selection pressure is demonstrated [92]. This technology has converted a natural system into a circuit which we can design artificially for in vivo evolution. However, escapees which have a mutation in their sensor or undesired sites enabling a faster growth compared to other cells with greater metabolic burden is a great challenge to this system.

Currently, three approaches are proposed to eliminate the escapees from the library. One is through in vitro compartmentalisation [93]. By encapsulating single-cell into microdroplet, each cell is being isolated. As there is no competing strain sharing the same culturing medium in droplet, low producing strains will be removed, and the improved strains are enriched by measuring the production or titre in each compartment, usually through fluorogenic label. Another approach is through re-transformation of plasmid into fresh parent strains after a few enrichment cycles of serial dilution [92]. In this approach, only the mutation of the desired fragments will be preserved, hence eliminating the escapees with mutation occurred on the host genome. However, these approaches are not continuous. Human intervention is required in each step. The third approaches is through introducing a toggle selector/counter-selector marker, i.e., tolC into the genetic circuit, which eliminate the escapees through negative selection [94]. This approach has enhanced the continuity of the process, but reduces the efficiency of selection.

To deal with the escapees in continuous evolution process, a specific carbon source utilisation-based selection strategy has been proposed [95]. A carbon source utilising cassette is placed under the control of the biosensor of targeted metabolites, coupling the production of the metabolite to cell fitness, i.e., the more carbon metabolite produced by the cells, the better it grows. Avoiding lethal selection pressure such as antibiotics help to reduce the probability of escapees generation, as the occurrence of adaptation to non-exploitable carbon source is rarer than antibiotics stress [95]. Using this approach, strains with l-tryptophan production increased by 65% were successfully enriched.

Recently, another form of selection based on syntrophic interaction called syntrophic co-culture amplification of production phenotype (SnoCAP) was successfully demonstrated. It amplifies distinguishability of production level into growth phenotype through metabolic cross-feeding circuit [96]. In this system, target molecule auxotroph sensor strain and target molecule secreting secretor strain which is an auxotroph to an orthogonal molecule secreted by sensor strain, are required. Mutant library of secretor strain is produced, and both sensor strain and secretor strain are co-cultured in in vitro compartment. Selection is conducted based on the final sensor-to-secretor ratio varied due to the genotype diversity of secretor strain. Although continuous selection was not performed in this study, it can be achieved by employing droplet-based cultivation system (refer next section).

## Equipment maintaining continuous culture

**Fig. 3:**
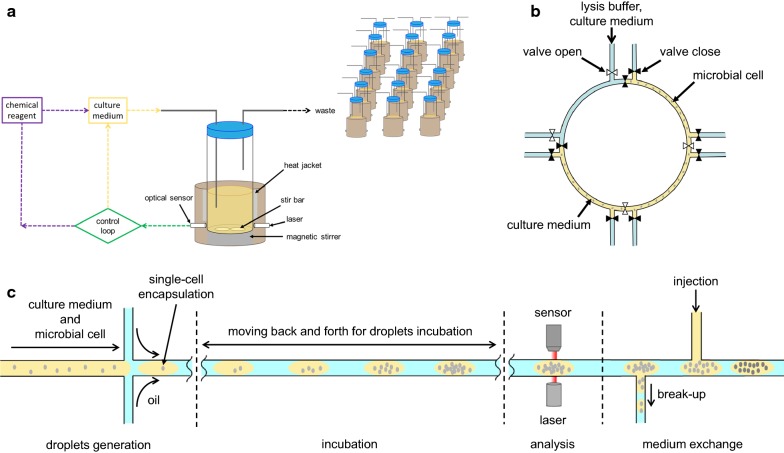
Equipment maintaining continuous culture. **a** In-vial continuous cultivation system. Each vial has an independent control logic, enabling parallel and compartmentalised continuous cultivation in macroscale. The whole system is designed to be programmable, empowering tailor-made continuous cultivation to fulfil the needs of each laboratories. **b** Microfluidic-based continuous cultivation system. Due to the scale of this system, single-cell analysis has been made possible, removing the barrier set by bulk analysis. Automation without human intervention can be achieved by using programmable parts, e.g., programmable syringe pumps. **c** Droplet-based continuous cultivation system. Almost all the operations involved in continuous cultivation can be performed automatically in this system in compartments, thus achieving rapid enrichment and high-throughput culturing system

The question remained in in vivo continuous evolution is, how to avoid the intervention of human in the culturing process. Conventionally, microbial cell cultivation and evolution depends on manual dilution culture in flask, which the root can be traced back to Louis Pasteur, the first microbiologist succeed in microbial cell cultivation. LTEE is a good example of microbial cell evolution based on manual dilution culture [76, 97, 98]. Great amount of effort, patience, and labour cost are involved in the whole experiment. As introduced at the beginning of this review, this is impractical for large, complex pathway optimisation for chemical production. Table 3 summarised various equipment maintaining continuous culture, while Fig. 3 shows some of the modern equipment. Miniaturisation of equipment can be observed along the history of continuous culturing system development, due to the requirements of parallelisation and compartmentalisation for high-throughput application or single-cell cultivation. Regardless of the size, partial or full automation has been achieved. Details of these technologies will be discussed in this section.

**Table 3:** Equipment maintaining continuous culture to sustain continuous evolution of microbial cell factories

System	Volume	Throughput	Modularisation	Control system	Detection system	Detection limit	Fluid manipulation	Operating duration without intervention	Principle	Ref
Flask	ml-l	Single	No	N/A	N/A	Bulk	manual	48 h	Shaken in shaker incubator to ensure well gas exchange. Dilution is performed when required	N/A
Chemostat	l	Single	No	Flow rate	N/A	Bulk	active pump	Several days	Continuous dilution is achieved by continuous flow of liquid medium in and out of the vessel	[99–101]
In-vial continuous cultivation system	< 15 ml	Several vials	Yes	Inhibitor concentration, cell concentration, stirring speed, temperature^a^	Optical density, fluorescence	Bulk	Passive pressure, millifluidic^a^, valve	24 h (Continuous cultured for 144 h)	Dilution triggered by defined parameter, while the liquid flow out by passive pressure. Parallelisation is achieved through miniaturisation and parallel control of multiple vials. However, biofilm formed in the vials and human intervention is required every 24 h	[103–105, 109]
Microfluidic-based continuous cultivation system	nl-ml	6 channels	No	Inhibitor concentration, cell concentration	Optical density, microscopy	Single-cell	microfluidic, valve	> 500 h	Continuous cultivation by circulating the culture around microfluidic channel. Biofilm is removed and the culture is diluted by frequently flushing a segment of the channel with lysis buffer and culture medium	[113, 114]
Droplet-based continuous cultivation system	nl-μl	~ Thousands of droplets	Yes	Inhibitor concentration, cell concentration, temperature	Optical density, fluorescence	Single-cell	Millifluidic, valve	Months	Encapsulation of microbial single-cell into droplet, and exchange medium through droplet breaking and pico-injection	[123, 124]

^a^These features are available in eVOLVER

### Flask cultivation

Flask cultivation is the oldest form of microbial cell cultivation, which is still a common practice in laboratory. Microbial cells are inoculated in sterile liquid medium in flask, and the flask is usually shaken in shaker incubator to ensure rich oxygen supply in the flask. This system is relatively simple. However, microenvironment fluctuation occurs when the essential nutrients in the flask is depleting. Furthermore, manual operations are required for each cycle of dilution, rendering microbial cell cultivation a time-consuming process.

### Chemostat and turbidostat

The invention of chemostat is a milestone in the history of microbial cell cultivation [99–101]. Although it is unintended, automation is achieved by open-loop control system through continuously replacing culture medium to maintain the nutrient in it. A culturing system with a closed-loop control system was later introduced as turbidostat [102]. Unlike chemostat which continuously diluting the culture at a fixed rate, turbidostat constantly monitors the optical density of the culture, and dilutes it when the optical density exceeds a predetermined threshold value or at a predefined point of time. This system enables a more robust automation for continuous evolution. To date, most of the systems proposed are the variants of chemostat or turbidostat.

### In-vial continuous cultivation system

Based on the design of turbidostat, a microbial selection device for in vivo continuous adaptive evolution called morbidostat was proposed [103, 104]. The growth rate of microbial cell is maintained by inhibitor, i.e. antibiotics, rather than dilution. Inhibitor is added into cell suspension only when the concentration exceeded predefined concentration and the growth rate is positive. Modifications were made on the modules to improve the precision of the system [105–108], but the major breakthrough in morbidostat was made in 2018, with the development of an equipment for continuous cultivation and evolution, eVOLVER (Fig. 3a) [109]. eVOLVER is a system well-balance the trade-off between controllability and throughput. Each ‘sleeve’ is independent, thus enables parallel experiments, increasing the throughput while maintaining the controllability of bioreactors. Instead of conventional fluid control module using pump and passive control such as pressure, a millifluidic was used in eVOLVER to ensure precise fluid manipulation.

However, a great challenge for all the chemostat, turbidostat and morbidostat in macroscale is the formation of biofilm. Biofilm unavoidably forms in all nutrient sufficient medium [110], while scale effect in in-vial continuous cultivation system worsen the condition [111]. The formation of biofilm will not only interfere the function of device, but also dominating the dilution of the culture [112]. Although automation is achieved for the dilution, culture exchange, and selection pressure introduction, human intervention is required for dilution at a larger scale and vial exchange is required every 24 h to prevent biofilm formation. Moreover, the cell conditions are measured in bulk, ignoring the heterogeneity of cells, leaving some important information unretrieved.

### Microfluidic-based continuous cultivation system

The small volume of microfluidic channels and automatic control of micropumps has contributed in the prevention of biofilm formation by periodic flushing of lysis buffer and culture medium in culturing channels (Fig. 3b). Furthermore, by culturing microbial cells in a microscale culturing system, the microbial cells can be analysed at single-cell level, nicely address the limitation of bulk analysis in conventional culturing system. Successful demonstration of long term culturing in a microchemostat for up to 500 h has encouraged the development of microfluidic-based microbial cell culturing system [113, 114]. Scale effect exhibited in mesoscale system has not only increased reaction rate in microfluidic system, but also improved some important parameters, e.g., oxygen transfer rate which is important to microbiology. In 1 ml working volume-turbidostat on-chip, oxygen transfer rate as high as 0.025 s^−1^, low mixing time and high control precision are achieved [115].

While single-cell analysis was made possible in microfluidic device, the culture is shared between all the microbial cells in the system. Risk of losing the information of slow growing but high production strain remains. Moreover, contamination occurs in devices when they are not properly designed. Contaminant might be trapped in the structure in the channel.

### Droplet-based continuous cultivation system

An improved method to support long-term in vivo continuous evolution in a compartmentalised system was proposed based on the study of André Lwoff [116]. Droplet encapsulation or in vitro compartmentalisation has offered various benefit besides single-cell analysis, i.e., isolation and enrichment of slow growing but high producing samples [93]. Taylor’s diffusion (refer [117]) and its consequence contamination can also be eliminated, while long-term cultivation is made possible with the invention of on-chip droplet formation [118], pico-injection [119, 120], droplet coalescence [121], and breakup module [122].

Millifluidic droplet analyser (MDA) is the first droplet-based continuous cultivation machine [123]. Growth of *E. coli* and minimal inhibitory concentration (MIC) for cefotaxime were measured using fluorescence signal. However, a junction is used for droplet formation, reducing its flexibility and the possibility for modularisation. On the other hand, a droplet-based cultivation system with on-chip droplet generation and detection modules has been proposed (Fig. 3c) [124]. The idea was materialised into a device called microdroplet microbial culture (MMC) system. Modularisation is achieved by using microfluidic chips in both droplet formation and analysis modules. Analytical module and droplet generation chips in MMC system are customisable, providing great flexibility to the system.

## Case studies of autonomous in vivo continuous evolution

Despite of various methods have been developed for in vivo continuous evolution, the applications are still an uncommon practice, due to the interdisciplinary technical requirements. Here, we discuss the applications of autonomous in vivo continuous evolution by interrelating the three aspects. The examples covered are listed in Table 4.

**Table 4:** Applications of in vivo continuous evolution in metabolic pathway evolution for chemical production

Description	In vivo genotype diversification	Fitness-coupled stress selection	Equipment for continuous cultivation	Advantages	Disadvantage	Effect	Refs.
Coupling ATP and lactate production to growth by engineering lactate production route as the sole anaerobic NADH oxidation route. Selection and enrichment were carried out during fermentation process in flask or fermentation vessels	Natural mutation	Metabolic evolution with LB or NBS medium	Flask or fermentation vessels	A relatively long evolution time is maintained. The setups are simple	Involvement of rational design in pathway construction can make the process difficult	Production of d-lactate increase from 865 $\pm$ 36 mmol l^−1^ to 1071 $\pm$ 2 mmol l^−1^	[83]
		Metabolic evolution with NBS medium with glucose and betaine (optional)				Production of l-lactate increase from 1228 $\pm$ 31 mmol l^−1^ to 1314 $\pm$ 48 mmol l^−1^	[85]
Coupling ATP and growth to alanine production and NADH oxidation. Selection and enrichment were carried out during fermentation process in flask or fermentation vessels		Metabolic evolution with NBS or AM1 medium with glucose				Production of l-alanine increase from 181 mmol l^−1^ to 1279 mmol l^−1^	[86]
D(-)-lactate hydrogenase fermentation pathway as the sole fermentative pathway coupled with the growth of strain. Selection and enrichment were carried out during fermentation process in flask or fermentation vessels		Metabolic evolution with NBS medium with 9% xylose				Production of ethanol increase from 250 mM to 950 mM	[87]
Coupling growth and glucose fermentation to NADH oxidisation pathway. Selection and enrichment were carried out during fermentation process in flask or fermentation vessels		Metabolic evolution with NBS or AM1 medium with glucose				Production of succinate increase from 108 mM to 699 mM while production of malate increases from 0 mM to 313 mM	[88, 89]
Maltose hydrolase is coupled to couple cell growth to tryptophan production. Selection and enrichment were performed in culture medium with maltose as the sole carbon source	Generation of library with ep-PCR followed by natural mutation	Artificial metabolite production/cell fitness coupling with maltose	Flask	Continuous evolution without the generation of escapees	Human intervention is required on every cycle of serial dilution	Increase in l-tryptophan titre from 0.5 mg l^−1^ OD_600_^−1^ to 5 mg l^−1^ OD_600_^−1^	[95]
EvolvR is performed to mutate rspE and rspL which is known to modify streptomycin resistance of bacteria. Serial culture was used to realise continuous culture	Genome-targeted mutagenesis with EvolvR	Artificial metabolite production/cell fitness coupling by culturing cells in selective media with xylose as sole carbon source	Flask	High-throughput mutant library generation	Human intervention is required on every cycle of serial dilution	16,000-fold increase in fraction of the population resistant to spectinomycin	[41]
SCRaMbLE was induced for 4 h and the cells were plated on synthetic dropout medium without uracil. Then, 87 coloies were picked at random and grown in selective medium with xylose as sole carbon source. The growth rate was monitored for 5 days	Recombination-based mutagenesis with SCRaMbLE	Artificial metabolite production/cell fitness coupling by culturing cells in selective medium with xylose as sole carbon source	Flask	High-throughput mutant library generation	Human intervention is required on every cycle of serial dilution	Growth rate increase from 0.18 h-1 to 0238 h-1 in culture medium with xylose as the sole carbon source.	[69]
dnaQ PE mutant library is generated and transfected into host cells to generate diversity in host cell. Under selection pressure, only the offspring with adaptive mutation can survive. These mutants can be used in biofuels production	Modified natural mutagenesis with dnaQ proofreading element (PE) mutant library	*n*-butanol	Flask	Continuous genotype diversification is achieved by transfected PE mutant	Human intervention is required on every cycle of serial dilution. Limited to specific strain	100-fold increase in survival rate in 2% *n*-butanol compared to wild type after 18 transfers.	[125]
		Acetate				eightfold increase in survival rate in 0.1% acetate compared to wild type after 12 transfers.	
PACE was performed to evolved the product protein aspartate kinase III, while l-lysine was used as a selection pressure for selection due to its inhibitory properties to aspartate kinase III	Modified natural mutagenesis with dnaQ926	l-lysine	Chemostat	Continuous evolution without any human intervention	Limited to specific strain which can be infected by bacteriophage	Absolute activity with 50 mM of lysine increase from 0.1 to 0.3; Less than 20% drop in relative activity when inhibited by 100 mM lysine	[130]

The most commonly used in vivo genotype diversification strategy is natural mutagenesis under selection pressure and modified natural mutagenesis. These are the simplest approach with the least experimental setups. In the studies of metabolic evolution [83–89], mutagenesis and enrichment are achieved through fermentation of products in flask or fermentation vessels, greatly reduce the complexity of the system. However, rational design is involved in metabolic pathway construction, increasing the hurdle of this technology. More effective in vivo genotype diversification approaches, e.g., EvolvR [41] and SCRaMbLE [69] were employed in some cases in in vivo continuous evolution to produce a diverse mutant library for selection. However, the genotype diversification in this approach was triggered only once in the lifetime.

Genome replication engineering assisted continuous evolution (GREACE) is the first demonstration to couple mutagenesis with selection [125]. DnaQ mutant library was transfected into *E. coli* to continuously trigger mutagenesis while the mutants generated are being selected under selection pressure containing in the same flask, i.e., toxic level of *n*-butanol and acetate. The improved strain can be obtained at a shorter time by synchronising in vivo genotype diversification and natural mutation.

Automated continuous culturing system was introduced in in vivo continuous evolution by phage-assisted continuous evolution (PACE) [126]. Unlike other systems, gene of interested are encoded in M13 bacteriophage while the mutagenic (mutagenesis plasmid) and selective (accessory plasmid) factors are harboured by *E. coli*. When the bacteriophage infected *E. coli*, mutagenesis of bacteriophage will be triggered by *E. coli* mutagenesis plasmid. Only those mutated bacteriophages which induce protein III (pIII) production will be released, realising the purpose of screening. The mutation rate can be further increased by mutators dnaQ926, umuC, umuD’ and recA730. Various proteins have been successfully evolved by this system [127–130].

The examples described above aimed to optimise the chemical production metabolic pathways of microbial cell factories through in vivo continuous evolution. Attempts were made to couple all the aspects in in vivo continuous evolution to increase the speed to improve microbial cells. However, to our knowledge, successful demonstration of in vivo continuous evolution integrating the modern technologies of all the aspects have not been reported. This might be due to the difficulties posed by the highly interdisciplinary requirements.

## Conclusions

Nature has always had an answer to process optimisation. By improving and mimicking nature’s system, an optimisation outcome better than any other system can be created. The development and integration of in vivo genotype diversification, fitness-coupled selection pressure, and equipment maintaining continuous culture is the key to realise in vivo continuous evolution, which has not been achieved before, due to the parallel development of profession in the past.

In vivo genotype diversification is the first step in in vivo continuous evolution to maximise the gene combination to cover the maximum space in the gene combinatorial space. Technology developed based on exploitation of nature existing system such as *Pol*III and bacteriophage, mutagenesis induction in microbial cell factories, utilisation of engineered DNAP system in cell to target a targeted fragment of gene. These systems are further improved by introducing mutagenesis at a high precision from two approaches, genome-targeted mutagenesis system and recombination-based mutagenesis system, both utilising viral systems.

The mutant library is then selected using fitness-coupled selection pressure, performed in equipment maintaining continuous culture. Selection pressure has not only promoted the survival of the fittest, but also the evolution to a better strain adapted to the microenvironment, in order to dominate the community. Various strategies to evolve microbial cell factories under selection pressure are introduced.

In order to maintain continuous evolution to mimic the continuity of the natural environment, automation technology has been brought into the perspective. Despite of being widely utilised in industry, chemostat and turbidostat have a relatively simple mechanism with large volume, making parallelisation a difficult process. Open-source hardware technology has accelerated the development of micro-cultivation system such as morbidostat, microfluidic-based cultivation system and droplet-based cultivation system, by international and interdisciplinary collaboration, have provided a robust platform for rapid evolution and selection. However, it is a great regret that these technologies are mostly used in studying antibiotic resistance evolution, even though they are good platforms for metabolic pathway in vivo continuous evolution for chemical production. Only with these continuous culturing technologies, that the study of genotype–phenotype relationship was made possible, open up the path to a better understanding on how genotype properties of microbial cells have linked to the phenotype expression, i.e., metabolite (chemical) production, to enable the rapid development of novel molecular biological tools for in vivo continuous evolution in the recent years. More modules for various platforms, e.g., absorbance-activated droplet sorting (AADS) technology for droplet-based continuous culturing system, have been developed to increase the detection precision and accuracy of the device, to enhance the speed to detect possible strain [131].

The advent of Turing learning machine [132] has provided a powerful tool to predict metabolic pathway in organisms [133]. This tool has also led to the optimisation of metabolic pathway [134]. Further improvement in computing power [135–137] will provide a greater platform to pathway optimisation. With these technological improvements, it is reasonable to predict that stoichiometric pathway optimisation of large, complex pathway might become possible. However, it is important to note that big data analysis and optimisation rely on what we have learned in the past, scripted as knowledge. Unfortunately, with our limited knowledge in metabolic pathways as input, it is still a difficult task to precisely predict the possible outcome of genetic modification by rational design. In this context, in vivo continuous evolution is still a promising tool in the future to optimise chemical production pathway. At the same time, both genotypic and phenotypic data collected as a result of mutation will further assist rational design of microbial cell factories.

## Data Availability

Not applicable.

## Abbreviations

AADS: absorbance-activated droplet sorting
ATP: adenosine triphosphate
Cas: CRISPR associated
CRISPR: clustered regularly interspaced short palindromic repeats
CTP: cytidine triphosphate
DNA: deoxyribonucleic acid
DNAP: deoxyribonucleic acid polymerase
DSB: double-strand break
ep: error-prone
GREACE: genome replication engineering assisted continuous evolution
GTP: guanosine triphosphate
HDR: homology-directed repair
HR: homologous recombination
indels: insertions and deletions
IDP: isopentenyl diphosphate
LTEE: long-term evolution experiment
MAA: mycosporine-like amino acid
MAGE: multiplex automated genome engineering
MDA: millifluidic droplet analyser
MIC: minimal inhibitory concentration
MMC: microdroplet microbial culture
MMEJ: microhomology-mediated end joining
nCas9: nickase CRISPR associated protein 9
NADH: nicotinamide adenine dinucleotide hydride
NHEJ: non-homologous end joining
NRPS: nonribosomal peptide synthase
PACE: phage-assisted continuous evolution
PCR: polymerase chain reaction
PKS: polyketide synthase
pol: polymerase
*Pol*I: DNA polymerase I
recβ: β recombinase
RNA: ribonucleic acid
RNAP: ribonucleic acid polymerase
SCRaMbLE: synthetic chromosome rearrangement and modification by loxP-mediated evolution
SnoCAP: syntrophic called syntrophic co-culture amplification of production phenotype
ssRNA: single-stranded RNA
TaGTEAM: targeting glycosylases to embedded arrays for mutagenesis
TCA: tricarboxylic acid
TP: terminal protein
μTAS: micro-total analysis system
